# Supplementary material for: Single-Nucleotide Polymorphisms in the Thioredoxin Antioxidant System and Their Association with Diabetic Nephropathy in Slovenian Patients with Type 2 Diabetes—A Preliminary Study
Source: Int J Mol Sci. 2025 Feb 20;26(5):1832. doi: 10.3390/ijms26051832 (PMC11899783; doi:10.3390/ijms26051832)
Supplement: Supplementary file 1 [file ijms-26-01832-s001.zip › ijms-3427678-supplementary.pdf]

SUPPLEMENTARY DATA

Supplementary Table S1. Association between Txn2_rs8140110 genotypes and some biochemical and clinical variables

TXN2_rs8140110	TT (N=14)	TC (N=158)	CC (N=725)	p value
**Fasting glucose [mmol/l]**	8.60 [7.93-10.23]	8.45 [7.23-9.90]	8.20 [6.80-9.70]	0.15 [a]
**HbA1c [%]**	7.45 [7.15-7.97]	7.60 [7.12-8.30]	7.50 [6.90-8.40]	0.20 [a]
Arterial hypertension				
**Yes**	13 (92.9%)	138 (87.3%)	640 (88.3%)	0.82 [b]
**No**	1 (7.1%)	20 (12.7%)	85 (11.7%)	
Statin use				
**Yes**	9 (64.3%)	115 (72.8%)	574 (79.2%)	0.10 [b]
**No**	5 (35.7%)	43 (27.2%)	151 (20.8%)	
**metformin**				0.53 [b]
**Yes**	4 (28.6%)	51 (32.3%)	264 (36.4%)	
**No**	10 (71.4%)	107 (67.7%)	461 (63.6%)	

Legend: [a] Kruskal-Wallis TEST was used; [b] Chi-square test was used. The value of $p < 0.05$ was considered statistically significant.

Supplementary Table S2. Association between TXNIP_rs7212 genotypes and some biochemical and clinical variables

TXNIP_rs7212	CC (N=2)	CG (N=53)	GG (N=842)	p value
**Fasting glucose [mmol/l]**	7.65 [7.53-7.78]	9.00 [7.50-10.20]	8.30 [6.90-9.80]	0.27 [a]
**HbA1c [%]**	7.65 [7.47-7.83]	7.80 [7.00-8.50]	7.50 [6.90-8.30]	0.29 [a]
Arterial hypertension				
**Yes**	2 (100.0%)	48 (90.6%)	741 (88.0%)	0.86 [b]
**No**	0 (0.0%)	5 (9.4%)	101 (12.0%)	
Statin use				
**Yes**	2 (100.0%)	44 (83.0%)	650 (77.2%)	0.64 [b]
**No**	0 (0.0%)	9 (17.0%)	192 (22.8%)	
metformin				0.15 [b]
**Yes**	2 (100.0%)	20 (37.7%)	297 (35.3%)	
**No**	0 (0.0%)	33 (62.3%)	545 (64.7%)	

Legend: [a] Kruskal-Wallis TEST was used; [b] Fisher Exact test was used. The value of $p < 0.05$ was considered statistically significant.

Supplementary Table S3. Association between TXNRD2_rs1548357 genotypes and some biochemical and clinical variables

TXNRD2_rs1548357	CC (N=84)	CT (N=388)	TT (N=425)	p value
**Fasting glucose [mmol/l]**	8.25 [6.57-9.62]	8.40 [6.90-9.80]	8.10 [6.90-9.80]	0.68 [a]
**HbA1c [%]**	7.40 [6.97-8.12]	7.60 [7.00-8.40]	7.50 [6.90-8.30]	0.44 [a]
**Arterial hypertension**				
**Yes**	72 (85.7%)	338 (87.1%)	381 (89.6%)	0.41 [b]
**No**	12 (14.3%)	50 (12.9%)	44 (10.4%)	
**Statin use**				
**Yes**	68 (81.0%)	295 (76.0%)	333 (78.4%)	0.54 [b]
**No**	16 (19.0%)	93 (24.0%)	92 (21.6%)	
**Metformin use**				0.0736 [b]
**Yes**	36 (42.9%)	147 (37.9%)	136 (32.0%)	
**No**	48 (57.1%)	241 (62.1%)	289 (68.0%)	

Legend: [a] Kruskal-Wallis TEST was used; [b] Chi-square test was used. The value of $p < 0.05$ was considered statistically significant.
